# Intrathyroidal Clear Cell Tumor of Parathyroid Origin with Review of Literature

**DOI:** 10.1155/2016/7169564

**Published:** 2016-11-27

**Authors:** Daniela Pirela, Daniela Treitl, Siba El Hussein, Robert Poppiti, Thomas Mesko, Alex Manzano

**Affiliations:** ^1^Mount Sinai Medical Center, Internal Medicine Department, 4300 Alton Road, Miami Beach, FL, USA; ^2^Mount Sinai Medical Center, Surgery Department, Miami Beach, FL, USA; ^3^Mount Sinai Medical Center, Pathology Department, Miami Beach, FL, USA; ^4^The Thyroid, Parathyroid and Pituitary Center for Miami, Internal Medicine Department, Miami Beach, FL, USA

## Abstract

Water-clear cell adenoma (WCCA) of the parathyroid gland is an exceedingly rare neoplasm. To date, 17 cases have been reported in the literature, with only one of them being intrathyroidal. Here we report a case of a 34-year-old woman who presented for evaluation of a goiter and was found to have a thyroid nodule and abnormal thyroid function tests (TFT). Fine needle aspiration biopsy of the nodule revealed thyroid follicular cells without atypia and subsequent Afirma® Gene Expression Classifier (GEC) testing results were suspicious for malignancy. As a result, the patient underwent a right thyroid lobectomy and isthmusectomy. Histological sections revealed an intrathyroidal nodule consistent with a clear cell neoplasm of parathyroid origin. The histologic appearance together with the immune profile was diagnostic of WCCA, with diffuse positivity for GATA3, focal weak positivity for parathyroid hormone, and negativity for PAX8, thyroglobulin, TTF1, synaptophysin, chromogranin, and S100p. Our study focuses on the clinical presentation, current management strategies, and review of the available literature surrounding this rare diagnosis. The ultimate goal is to help endocrinologists and surgeons establish a foundational treatment plan for intrathyroidal clear cell tumor cases.

## 1. Introduction

Water-clear cell hyperplasia (WCCH) and water-clear cell adenoma (WCCA) are rare entities [1, 2], with the first case of WCCH being published in 1934 by Albright et al. [3] and the first WCCA case not until 1994 by Kovacs et al. [4]. WCCA has been defined as a solitary mass, with lack of lobulation and lack of fat within the lesion [5]. Similar to WCCH, WCCA are composed of cells with abundant clear to foamy or granular cytoplasm and mild nuclear pleomorphism [4–7]. It is more frequent in patients in their 40s to 50s with no gender predilection [2].

Histopathology findings of clear cell changes can be present in other head and neck tumors and metastatic entities including thyroid neoplasms, salivary gland neoplasms, paragangliomas, and metastatic renal cell carcinomas [1]. Consequently, this becomes a diagnostic challenge especially on fine needle aspiration (FNA) or frozen section evaluation [1]. The right clinical scenario for WCCA is not always straightforward. Despite relatively high PTH levels, serum calcium is not particularly elevated, consistent with low endocrine activity of WCCA [2, 4, 8–10]. Adding to the clinical challenges of identifying or classifying this rare malignancy, there is a lack of literature to reference for WCCA specific treatment indications.

The literature suggests that parathyroid ectopia occurs in 4–20% of patients as a consequence of abnormal migration during embryogenesis or secondary to acquired migration. Ectopic inferior parathyroid is more common than superior parathyroid ectopia, possibly due to a longer and more variable embryological migration of inferior parathyroid glands. Intrathyroidal parathyroid adenoma location varies from 1.4 to 6% [11, 12]. In published reports, parathyroid WCCA has been reported in 17 cases to date, with only one case where the tumor was intrathyroidal [13]. Here we describe the second case of an intrathyroidal clear cell adenoma of parathyroid origin, with the goal of informing and providing a reference for better clinical judgment in WCCA cases.

## 2. Case Presentation

**Figure 1:**
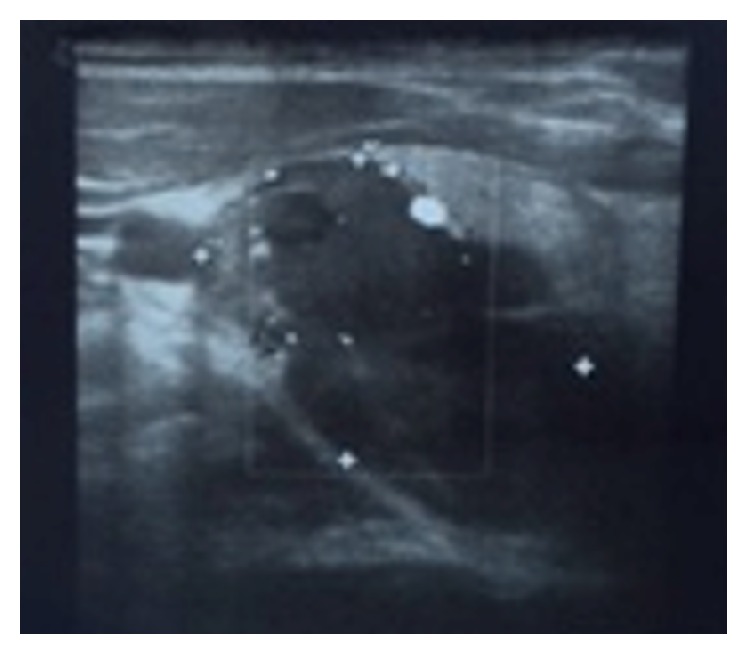
Thyroid ultrasound, large dominant complex 3.6 × 2.6 × 3.3 cm thyroid nodule with regular borders and peripheral vascularity in the right lobe.

The patient is a 34-year-old female with a goiter who presented initially to the clinic after she was found to have a thyroid nodule on ultrasound of the neck and abnormal thyroid function tests (TFT). The patient had a family history significant for having thyroid cancer and Graves' disease. Although the patient did not claim any history of radiation exposure, she was concerned about recent weight gain, hair loss, and increased sweating, but denied any voice change, neck pain, or dysphagia. Vital signs were within normal limits, and a nontender goiter was appreciated during the neck exam. The remainder of the physical exam was unremarkable including neurological reflexes. She did not have symptoms of hyperparathyroidism, and her calcium level was 9.3 mg/dL (8.5–10.1). PTH was not measured due to lack of the clinical scenario of hyperparathyroidism. Thyroid ultrasound showed a large dominant complex 3.6 × 2.6 × 3.3 cm thyroid nodule with regular borders and peripheral vascularity in the right lobe (Figure 1).

TFT was relevant for TSH 0.305 (0.358–3.740 uiu/mL), free T4 1.16 (0.76−1.46 ng/dL), free T3 3.2 (2.18−3.98 pg/mL), T3 total 102 (71−180 ng/dL), thyroglobulin 48 (2–35 ng/mL), thyroglobulin ab (antibody) 0.9 (0.0−0.9 IU/mL), thyroid peroxidase ab 9 (0−34 IU/mL), and thyroid stimulating immunoglobulin 31 (0−139%). These findings were suggestive of subclinical hyperthyroidism. Fine needle aspiration of the nodule revealed cellular evidence of a follicular lesion with thyroid follicular cells without atypia in a background of minimal colloid on Giemsa stain. Afirma GEC testing result was suspicious; therefore, the patient underwent a right thyroid lobectomy and isthmusectomy. After the procedure, the calcium level trended down to 7.9 before discharge.

## 3. Pathological Examination

**Figure 2:**
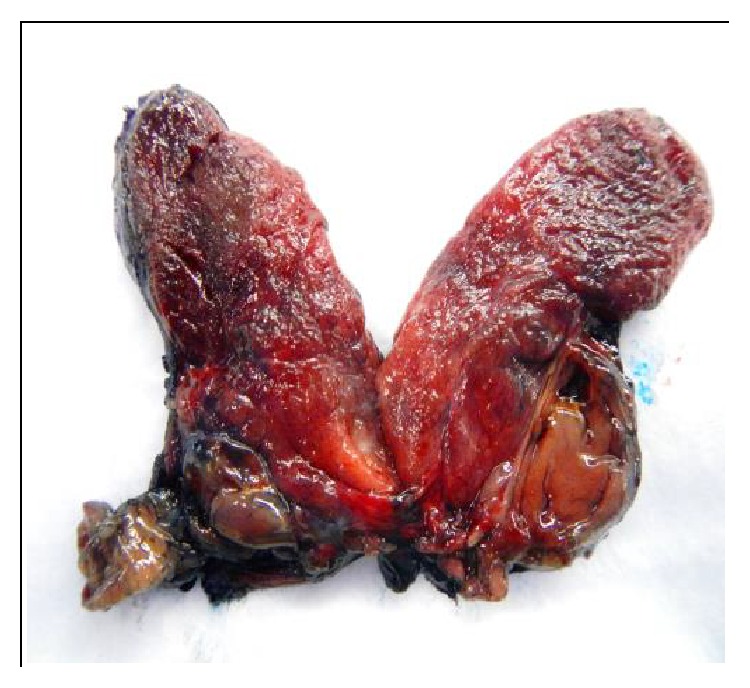
Circumscribed tan intrathyroidal 2.5 × 1.7 × 1.5 cm mass abutting the margin grossly.

**Figure 3:**
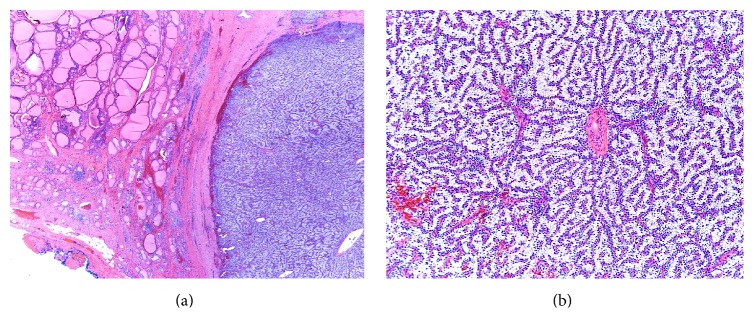
(a) Normal thyroid tissue on the upper left corner, (b) parathyroid neoplasm on the water-clear cell adenoma.

Gross examination revealed a thyroid lobe and isthmus weighing 27.7 grams (Figure 2). The thyroid lobe measured 6 × 4 × 2 cm and the isthmus measured 1 × 1 × 1 cm. The specimen was bisected to reveal a circumscribed, tan nodule measuring 2.5 × 1.7 × 1.5 cm abutting the margin grossly. Microscopic examination of the intrathyroidal nodule revealed a circumscribed encapsulated clear cell neoplasm, composed exclusively of uniform monotonous cells with abundant, clear finely vacuolated cytoplasm. The nuclei showed minimal atypia, with finely stippled chromatin. The cells were aggregated in nests and were separated by thin fibrovascular septae with no intervening fat. No necrosis or significant mitotic activity was identified (Figure 3).

**Figure 4:**
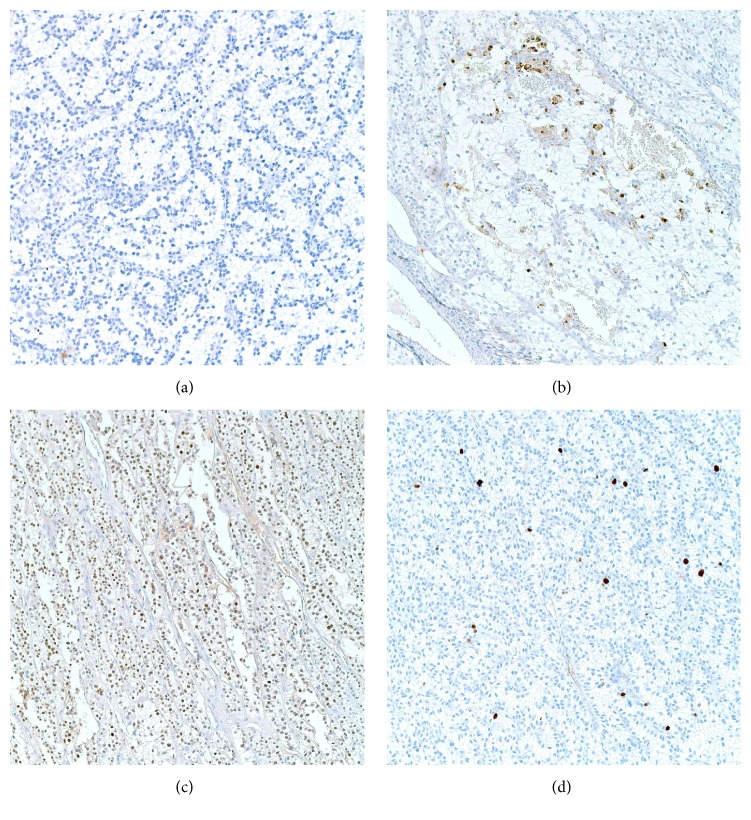
(a) Thyroglobulin stain negative. (b) Focally weakly positive parathyroid hormone stain. (c) Negative PAX8 stain. (d) KI 67 showing low mitotic rate.

Immunohistochemistry revealed diffuse positivity for GATA3 with weak focal positivity for parathyroid hormone, and negativity for PAX8, thyroglobulin, TTF1, synaptophysin, chromogranin, and S100p. Ki67 staining revealed a proliferative rate of less than 2%. Cytoplasmic accumulation of glycogen was confirmed by a positive periodic acid-Schiff (PAS) and negative diastase-PAS reactions. The architecture, cytology, and the immunoprofile supported the diagnosis of an intrathyroidal water-clear cell adenoma of the parathyroid (Figure 4).

## 4. Discussion

WCCA are exceedingly rare; our literature review identified 18 cases reported to this date worldwide including our case (Table 1) [1, 2, 4–10, 13–19]. Parathyroid adenomas are usually composed of chief cells or oxyphilic cells. In contrast, WCCA are composed of uniform monotonous cells with abundant, clear finely vacuolated cytoplasm thought to be derived from the Golgi apparatus or possibly from the endoplasmic reticulum [2, 4, 5, 18, 20]. In addition, FNA biopsy may be nonspecific and misleading [1, 20]. Consequentially, clinical and cytological findings make the diagnosis of WCCA challenging and neoplasms such as salivary gland tumors, paragangliomas, and metastatic renal clear cell carcinomas (ccRCC) should be considered in the differential diagnosis [1, 17].

Salivary gland tumors can show variable proportions of clear cells. Paragangliomas will show positivity for synaptophysin, chromogramin, and S100 protein stains in sustenticular cells with negativity for PTH [1]. Metastatic ccRCC to the thyroid is not uncommon in patients with a concurrent undiagnosed primary or with a remote history of ccRCC. Immune-histochemical reactivity with vimentin, PAX 8 transcription factor, and CD10 can be helpful. Renal cell carcinoma (RCC) antigen has been reported to be positive in parathyroid adenomas and does not exclusively identify ccRCC metastases. An immune profile of transcription factor 1 (TTF-1) and thyroglobulin (TGB) negative with positive carbonic anhydrase IX (CAIX) positive has been described as 100% sensitive and specific for metastatic ccRCC in the thyroid. TFF-1 positive, TGB positive, and CAIX negative support a primary thyroid neoplasia [1, 20].

Parathyroid FNA can have follicular formation, papillary fragments, and even colloid [1, 15]. Clinical history, tumor location, and high levels of PTH in the aspirated material support the diagnosis of primary parathyroid lesions. Negative TGB stain will differentiate parathyroid from thyroid lesions [1].

We report the second case of intrathyroidal parathyroid WCCA with the interesting asymptomatic presentation of a normal calcium level. Negativity for PAX8 ruled out ccRCC; negativity for chromogramin and synaptophysin ruled out paraganglioma. Negativity for TTF1 and thyroglobulin ruled out thyroid neoplasm. Finally, the presence of uniform cells with abundant clear finely vacuolated cytoplasm and positivity for GATA3, with weak focal positivity for parathyroid hormone, supported the diagnosis of WCCA of the parathyroid.

**Figure 5:**
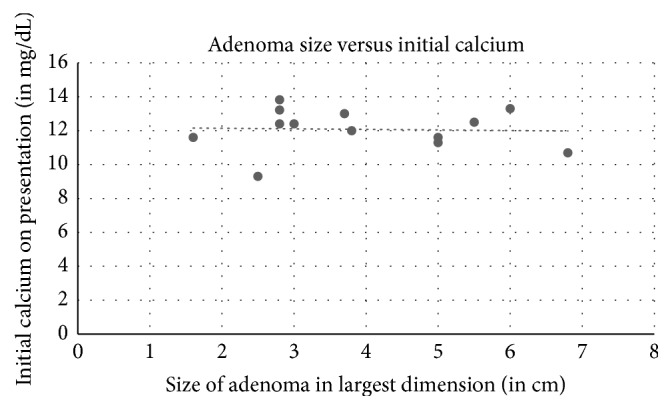
Size of adenoma on pathological evaluation correlation to initial calcium on presentation. Correlation coefficient −0.04.

**Figure 6:**
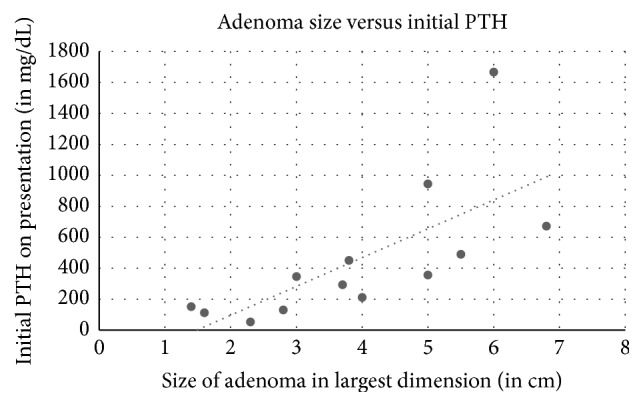
Size of adenoma on pathological evaluation correlation to initial PTH on presentation. Correlation coefficient 0.71.

**Figure 7:**
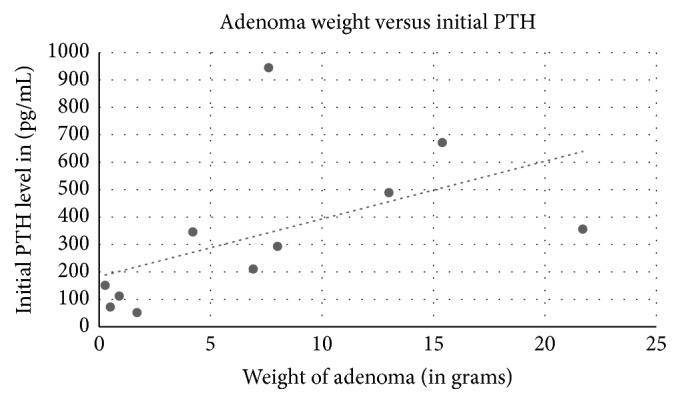
Weight of adenoma on pathological evaluation correlation to initial PTH on presentation. Correlation coefficient 0.53.

**Figure 8:**
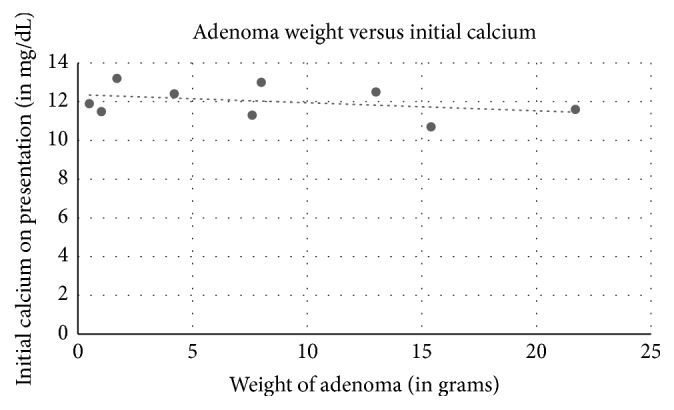
Weight of adenoma on pathological evaluation correlation to initial calcium on presentation. Correlation coefficient −0.37.

Among the 18 cases reviewed including ours, 11 were females and 7 were males (Table 1). The average age was 57 years old (18 to 81 years old). Ultrasound of the thyroid was used in 14 cases, 12 with positive results. Sestamibi scan was used in 12 patients: 7 were with positive findings and 5 were negative. Of the 4 cases that used FNA biopsy, one case was diagnosed as a colloid nodule, one as a follicular neoplasia of the thyroid, one as concomitant papillary thyroid cancer, and our case as thyroid follicular cells without atypia. Symptoms depended on the length and severity of PTH levels and hypercalcemia. Average calcium at initial presentation was 11.98 mg/dL. Five of the cases did not describe symptoms, one case mentioned an asymptomatic patient with normal PTH levels and elevated calcium [15], one patient developed pancreatitis [7], and other patients developed nephrolithiasis, bone loss and fractures, fatigue, and muscle cramps. One case had a history of neurofibromatosis 1 [17], and one case Multiple Endocrine Neoplasia 1 [4]. Three patients had both unspecified parathyroidectomy and thyroidectomy, 2 patients had thyroidectomy alone, and 12 patients had parathyroidectomy. Most lesions were extrathyroidal, with the average size in the greatest dimension of 3.9 cm and the average weight of 7.3 g. Our case, an asymptomatic patient with normal calcium levels and a small lesion, is consistent with the reported low endocrine activity of WCCA. [2]. Indeed, adenoma size seems to have no correlation to initial calcium levels in the 13 cases with reported adenoma size and initial calcium (Figure 5, correlation coefficient −0.04). However, there is a positive correlation between initial presenting PTH level and size of adenoma on pathological examination (Figure 6, correlation coefficient 0.71). There is also a positive correlation between initial presenting PTH level andweight of adenoma (Figure 7, correlation coefficient 0.53), while there is a weakly negative correlation between initial presenting calcium level and weight of adenoma (Figure 8, correlation coefficient −0.37).

Patients with symptomatic hyperparathyroidism should undergo surgery following the current guidelines [21]. Treatment of WCCA of the parathyroid remains surgical in nature with parathyroidectomy assisted by sestamibi scan and ultrasound, and in the case of intrathyroidal lesions, thyroidectomy.

Parathyroid carcinoma is rare and to our knowledge only one case of water-clear cell carcinoma (WCCC) of the parathyroid has been described [22]. Local invasion and, of course, metastases are part of the criteria to establish the diagnosis of carcinoma [6, 22].

## 5. Conclusion 

WCCA is a rare neoplasm that should be considered in the differential diagnosis of clear cell lesions of the neck. Parathyroid WCCA are typically asymptomatic, benign lesions with low endocrine activity and are mostly found outside of thyroid tissue, with occasional intrathyroidal localization. The goal of this study was to provide pathologists, endocrinologists, and surgeons with additional knowledge surrounding this rare entity and to increase diagnostic awareness. Symptomatic WCCA should be removed like other parathyroid adenomas. However, management of asymptomatic lesions is not a clear cut, as early diagnosis may prove difficult in the setting of the lack of knowledge of this disease with low endocrine activity. Here we highlight that adenoma size is correlated to initial PTH levels but not initial calcium levels and that larger lesions are more likely to be symptomatic and therefore may need closer followup. In asymptomatic patients, tumors with radiographically suspicious features or malignant behavior should be resected.

## Figures and Tables

**Table 1:** 

Literature	Age, sex	Diagnosis	FNA	Dimensions in cm; extrathyroidal unless specified	Weight	Symptoms	PTH level in pg/mL	Initial calcium in mg/dL	Treatment
Kovacs et al. 1994 [4]	48 M	US	NA	NA	NA	NA	43	10.8	Right hemithyroidectomy and total parathyroidectomy
Grenko et al. 1995 [5]	40 M	NA	NA	5 × 3 × 1.5	7.6 g	Fatigue, leg cramps	945	11.3	Parathyroidectomy
Bégueret et al. 1999 [14]	73 M	NA	NA	2.8 × 1 cm	NA	Nephrolithiasis	207	13.7	NA
Dundar et al. 2001 [13]	43 F	(+) US/(+) sestamibi scan	NA	6; intrathyroidal	NA	Fractures, cramps, fatigue	1667	13.3	Left total and right near total thyroidectomy
Kuhel et al. 2001 [15]	56 F	(+) US/(−) sestamibi scan	FNA colloid nodule	Right 2.8 Left 1.5	1.7 g 0.5 g	Asymptomatic	52	13.2	Parathyroidectomy, R thyroid lobectomy, and isthmectomy
Kanda et al. 2004 [2]	52 F	(+) US/(+) sestamibi scan	NA	6.8 × 2.8 × 1.9	15.4 g	Gastritis, nephrolithiasis	672	10.7	Parathyroidectomy
Prasad et al. 2004 [16]	40 F	(+) US	NA	3 × 1.5 × 1.0	4.2 g	Fatigue, cramps, weakness	346	12.4	Parathyroidectomy
Kodama et al. 2007 [17]	18 F	(+) US/(+) sestamibi scan	NA	5 × 3.3 × 3	21.7 g	Renal stones	356	11.6	Parathyroidectomy
Papanicolau-Sengos et al. 2011 [1]	64 M	(+) CT chest with contrast	Follicular neoplasm of the thyroid	4.7 × 3.5 × 1.7	NA	Asymptomatic	NA	NA	Parathyroidectomy
Chou et al. 2014 [7]	81 F	(+) US/(+) sestamibi scan	NA	3.8	NA	Hyper/pancreatitis	450	12.0	Parathyroidectomy
Bai et al. 2012 [18]	81 M	(−) US/(+) sestamibi scan	NA	4 × 2.5 × 1.6	6.91 g	NA	211	NA	Parathyroidectomy
Bai et al. 2012 [18]	55 M	(−) US/(−) sestamibi scan	Concomitant papillary thyroid carcinoma	1.4 × 0.8 × 0.6	0.27 g	NA	151	NA	Thyroidectomy, right neck lymph node dissection, and parathyroidectomy
Piggott et al. 2013 [8]	74 F	(+) US/(+) sestamibi scan	NA	5.5 × 2.5 × 2	13 g	Abdominal pain, constipation, lethargy	489	12.5	Parathyroidectomy
Ezzat et al. 2013 [10]	73 M	(+) US/(+) sestamibi scan	NA	3.7 × 3.5 × 1.7	8 g	NA	293	13.0	Parathyroidectomy
Ezzat et al. 2013 [10]	74 F	(+) US/(−) sestamibi scan	NA	1.6 × 1.2 × 0.3	0.9 g	NA	112	11.6	Parathyroidectomy
Tassone et al. 2014 [9]	54 F	(+) US/(−) sestamibi scan	NA	2.8 × 1.1 × 1.1	NA	Hyper/bone pain, depression, forgetfulness	130	12.4	Parathyroidectomy
Murakami et al. 2014 [19]	59 F	(+) US/(+) CT chest/(−) sestamibi scan	NA	NA; extrathyroidal	0.5 g	Renal stones, bone fracture	72	11.9	Parathyroidectomy
Current case	34 F	(+) US	Thyroid follicular cells without atypia	2.5 × 1.7 × 1.5 intrathyroidal	NA	Asymptomatic	NA	9.3	Thyroidectomy

NA: not available; (+): positive; (−): negative; sestamibi scan: sestamibi scan of the parathyroid; US: US of the thyroid.
